# Molecular and Cellular Mechanisms of Arthritis in Children and Adults: New Perspectives on Applied Photobiomodulation

**DOI:** 10.3390/ijms21186565

**Published:** 2020-09-08

**Authors:** Laura Marinela Ailioaie, Gerhard Litscher

**Affiliations:** 1 Department of Medical Physics, Alexandru Ioan Cuza University, 11 Carol I Boulevard, 700506 Iaşi, Romania; lauraailioaie@yahoo.com; 2 Ultramedical & Laser Clinic, 83 Arcu Street, 700135 Iaşi, Romania; 3 Research Unit of Biomedical Engineering in Anesthesia and Intensive Care Medicine, Research Unit for Complementary and Integrative Laser Medicine, and Traditional Chinese Medicine (TCM) Research Center Graz, Medical University of Graz, Auenbruggerplatz 39, 8036 Graz, Austria

**Keywords:** adults, juvenile, cytokines, immune, laser blood irradiation, low-level laser, systemic

## Abstract

Juvenile idiopathic arthritis and adult rheumatoid arthritis are two major groups with chronic joint pain and inflammation, extra-articular manifestations, and high risk of comorbidities, which can cause physical and ocular disability, as well as create great socio-economic pressure worldwide. The pathogenesis of arthritis manifested in childhood and adulthood is multifactorial, unclear, and overly complex, in which immunity plays an important role. Although there are more and more biological agents with different mechanisms of action for the treatment of arthritis, the results are not as expected, because there are partial responses or non-responsive patients to these compounds, high therapeutic costs, side effects, and so on; therefore, we must turn our attention to other therapeutic modalities. Updating knowledge on molecular and cellular mechanisms in the comparative pathogenesis of chronic arthritis in both children and adults is necessary in the early and correct approach to treatment. Photobiomodulation (PBM) represents a good option, offering cost-effective advantages over drug therapy, with a quicker, more positive response to treatment and no side effects. The successful management of PBM in arthritis is based on the clinician’s ability to evaluate correctly the inflammatory status of the patient, to seek the optimal solution, to choose the best technology with the best physical parameters, and to select the mode of action to target very precisely the immune system and the molecular signaling pathways at the molecular level with the exact amount of quantum light energy in order to obtain the desired immune modulation and the remission of the disease. Light is a very powerful tool in medicine because it can simultaneously target many cascades of immune system activation in comparison with drugs, so PBM can perform very delicate tasks inside our cells to modulate cellular dysfunctions, helping to initiate self-organization phenomena and finally, healing the disease. Interdisciplinary teams should work diligently to meet these needs by also using single-cell imaging devices for multispectral laser photobiomodulation on immune cells.

## 1. Introduction

Chronic arthritis is the most usual cause for joint pain, physical disability, and ocular invalidity worldwide. Juvenile idiopathic arthritis (JIA) and rheumatoid arthritis (RA) of the adult are two major groups with chronic joint inflammation, extra-articular manifestations, and high risk for comorbidities [1,2,3,4,5].

While in adults there are over 150 forms of chronic arthritis, in children, there are several dozen subtypes of the disease, but only juvenile polyarthritis with positive rheumatoid factor and the subtype of systemic arthritis also known as Still disease, which is more consistent with an autoinflammatory condition, have similar manifestations to adults [6].

Patients with systemic JIA require close keep under surveillance by a multidisciplinary team due to possible serious complications: macrophage activation syndrome, pericarditis, pulmonary hypertension, interstitial lung disease, infections, etc., which may be associated with increased mortality [7].

In the pathology of children, the oligoarticular manifestation is an entity that we do not find in the forms of rheumatic disease in adults and is characterized by often severe eye damage, localized growth disorders with elongation of a limb, and secondary posture disorders.

JIA is the type of chronic rheumatic disease that affects the child’s daily activities due to pain, joint swelling, morning stiffness, and locomotor and possibly ocular infirmities, which causes short-term and long-term disabilities, until adulthood and sometimes throughout life [8].

Treatment available for patients with chronic arthritis aims to reduce pain, maintain joint function, improve well-being, and prevent disability and associated comorbidities.

Pharmacological therapy usually includes non-steroidal anti-inflammatory drugs, intra-articular or systemic steroids, to which will be added disease-modifying anti-rheumatic drugs (DMARDs) and biological agents administered on time in the “window of opportunity” to prevent irreversible complications [2].

Early use of intra-articular steroid therapy, methotrexate, and biological agents introduced in recent decades have improved the prognosis of children with arthritis, but those with polyarticular form can have serious problems with active disease as adults. Most children with the JIA oligoarticular subtype may enter remission, but a small number progress to a persistent polyarticular form as adults.

Concerns have been raised about the use of biological agents that may increase the risk of cancer in patients with chronic arthritis.

Based on the severity of the disease, which evolves progressively, the patient with chronic arthritis can become an important burden for the family, but especially for the society, through the enormous costs of direct health care, social assistance, loss in education, productivity, and jobs.

The first goal of this review was to update knowledge on molecular and cellular mechanisms through a parallelism between special forms of chronic arthritis present in both children and adults, for an introspection into the pathogenesis of these diseases, in an attempt to reveal to researchers and clinicians the latest discoveries regarding new molecules and signaling pathways.

The second objective of this review was to raise awareness and send a signal to rheumatologists on the need to change the treatment paradigms for arthritis through innovative therapies to stop the perpetuation of the disease from childhood to adulthood, the side effects, the inefficiency in some cases, and the high current costs, in order to overcome this human and economic burden.

The third purpose was to promote light or laser therapies (photobiomodulation) as an important complementary and alternative method, which has become increasingly known around the world in recent decades for reducing pain and sometimes even eliminating the cause of the pain itself, for inducing early remission before common destructive changes in joints begin, in all arthritis forms.

Last aim but not least is to signal that photobiomodulation (PBM) and the single-cell live tracking technology of immune cell activities are ready to precisely target the signaling pathways and to find the answers to the complex interaction of the laser with the immune system, for “undoing” arthritis!

Seeking and developing new treatments to interact smoothly with the immune system both in children and adults to handle immune-mediated diseases that are becoming more and more complex is urgently needed.

JIA, formerly known as juvenile rheumatoid arthritis in the Anglo-Saxon literature, and chronic juvenile arthritis for French speakers, is a chronic immune-mediated inflammatory disease of unknown etiology and a complex genetic component that is defined according to the criteria of the International League of Associations for Rheumatology (ILAR) as inflammatory arthritis in one or more joints, which begins before the age of 16, persists for at least six weeks, and all other conditions that cause similar symptoms have been excluded [1,4].

A better understanding of the pathogenesis and the latest diagnostic tools are challenges for rheumatologists to update the classification. Based on ILAR criteria, there are seven main subgroups of JIA defined by clinical and laboratory data: systemic arthritis, rheumatoid factor (RF) polyarthritis—positive or negative, oligoarthritis (persistent or extensive), enthesitis-related arthritis (ERA), psoriatic arthritis (PsA), and a seventh category, undifferentiated arthritis, which includes those patients who do not fit any of the above forms of criteria [4,9].

### 1.1. RF-Positive Polyarticular JIA

RF-positive polyarticular juvenile idiopathic arthritis is defined by the existence of at least 5 inflamed joints over a period of 6 weeks, in the presence of RF found twice, at an interval of at least 3 months in the first 6 months after the onset of the disease. This category is considered clinically and biologically similar to adult RA by progressive destructive polyarticular manifestations in the knee, elbow, and foot.

In this case, arthritis in children is symmetrical and predominantly peripheral; it affects particularly small joints of the fingers and toes, but it may also affect the large joints of the knees, hips, ankles, and fist.

Other manifestations may include lower-level fever than in systemic form, rheumatoid nodules (tumors under the skin, most common in the elbow), anemia, and thrombocytosis.

Factors that determine disability include early age at onset, female gender, the presence of rheumatoid factor, and the presence of anti-cyclic citrullinated peptide antibodies (anti-CCP) [2,10].

As a peculiarity, it should be mentioned in children that it affects the temporomandibular joints and the upper cervical region (neck area).

Temporomandibular arthritis can limit mouth opening and discomfort in chewing. Arthritis of the neck area can cause instability or fusion of the cervical vertebrae, with a high potential for neurological injury of the spine to minor trauma.

Long-term studies show that the prognosis is severe in 50% of cases [11].

Most common complications are osteoporosis, vertebral collapse, dwarfism, pubertal delay, intercurrent viral, or bacterial infections in immunosuppressed children by disease or secondary to medication [12,13].

### 1.2. RF-Negative Polyarthritis

Rheumatoid factor negative polyarticular juvenile idiopathic arthritis is defined by inflammatory damage of 5 or more joints, in the first 6 months after onset, in the absence of RF [14].

According to some authors, this type of juvenile arthritis accounts for up to 30% of polyarticular forms of childhood, and in some cases, it has a rapidly disabling progression [15,16].

This category of polyarthritis is much more severe than oligoarthritis and is often associated with extra-articular manifestations that include salivary gland disease (Sjögren’s Syndrome), lymphadenopathy within Felty’s Syndrome, or juvenile vasculitis.

About 20% of this category of arthritis starts early, affects the female gender, to whom can be detected positive antinuclear antibodies (ANA), and in these cases, there is a high risk for iridocyclitis. The long-term functional prognosis of the disease is more severe than in the oligoarthritis subtypes, but it is better than in the RF-positive polyarticular JIA [2].

### 1.3. Systemic JIA

Systemic juvenile idiopathic arthritis (sJIA) is a subtype of the disease that occurs in childhood secondary to an immune disorder, which associates arthritis and systemic inflammatory symptoms [17].

sJIA is defined as arthritis accompanied or preceded by daily fever, with a minimum duration of 2 weeks, associated with the following extra-articular symptoms: erythematous rash, lymph node hypertrophy, hepato- and/or splenomegaly, and serositis [18].

The diagnosis is sustained by the prolonged fever for at least two weeks and is accompanied by two major criteria, or by one major criterion and two minor criteria. The major criteria are given by erythematous rash and arthritis. The minor criteria could be the following: generalized adenomegaly and/or hepatomegaly and/or splenomegaly; serositis; arthralgia lasting 2 weeks or more (in the absence of arthritis); and leukocytosis (≥ 15,000 /µL) and increased numbers of neutrophils.

sJIA is similar to Still’s disease in adults and for the correct diagnosis, the presence of fever is required alongside at least one major criterion; arthritis would no longer be necessary because, as in adults, it may be initially missing [19].

### 1.4. Enthesitis-Related Arthritis (ERA)

The enthesitis-related arthritis (ERA) has also been referred to by ILAR as an undifferentiated spondyloarthropathy, because the symptoms are mostly found in adulthood, but with a higher percentage of non-differentiated forms in children. In the current definition, an imagistic criterion was introduced by radiographic images or magnetic resonance images [20,21].

The diagnosis is supported by peripheral arthritis and enthesitis, or arthritis, or enthesitis, which are associated with ≥ 3 months of inflammatory back pain and sacroiliitis on X-ray or MRI images. Arthritis or enthesitis must persist for at least 6 weeks, to which are added the following symptoms: sensitivity of the sacroiliac joint; back pain, the presence of HLA-B27 antigen; previous acute uveitis (symptomatic); and a history of spondylarthritis in a first-degree relative [19,22,23].

Over the last decade, more and more evidence has been gathered suggesting that some of these categories appear to be quite homogeneous and are present both in children and adults; others are heterogeneous and cannot be better defined [24].

### 1.5. Psoriatic JIA

Psoriatic juvenile arthritis (psJIA) accounts for up to 10% of all JIA subtypes; it is a type of arthritis that affects both sexes and manifests itself in association with psoriatic skin lesions [25].

Psoriatic arthritis is defined within the ILAR classification as a persistent arthritis of more than 6 weeks and either the presence of a psoriatic rash or, in the absence of rash, at least 2 of the following minor criteria: first degree relative with psoriasis, nail pitting, onycholysis, dactylitis; the forms with positive RF and those associated with systemic manifestations are excluded. The peculiarity of psoriatic rheumatism is represented by the presence of ANA in more than 50% of cases and by its association with uveitis [4,25].

### 1.6. Oligoarticular JIA

Oligoarticular juvenile idiopathic arthritis, formerly referred to as pauciarthritis or juvenile rheumatoid arthritis with pauciarticular onset, is defined by the inflammatory involvement of one or less of five joints [2].

It is the most common subgroup in juvenile arthritis, accounting for about 50% of all cases. The oligoarticular JIA comprises two categories: the persistent form in which the number of inflamed joints remains the same throughout the disease, and the extensive form in which the number of active joints is five or cumulatively more, after the first 6 months onset [2,16].

A particular case is that of oligoarthritis as a more homogeneous entity observed only in childhood, with early onset and an association of positive antinuclear antibodies (ANA) [26], young age, and female gender, complicated with iridocyclitis [19,27,28,29].

### 1.7. Undifferentiated Arthritis

Undifferentiated JIA is a subtype of juvenile arthritis that does not exactly meet the criteria for the categories mentioned above, or it simultaneously meets several criteria for different subtypes of the disease [2].

The Paediatric Rheumatology INternational Trials Organisation (PRINTO) has a current project with the proposal to revise current JIA classification criteria after ILAR, using clinical evidence and common laboratory data available worldwide to classify those forms of chronic arthritis that are commonly encountered in children and that are the counterpart of childhood diseases observed in adults [19].

## 2. Molecular and Cellular Mechanisms of Systemic Arthritis

The pathogenesis of systemic arthritis manifested in childhood and adulthood is multifactorial, unclear, and very complex, in which the innate immunity plays an important role by activating neutrophils and macrophages, as well as the adaptive immunity, by increasing the percentage of pro-inflammatory cytokines: interleukin (IL)-1β, IL-6, IL-18, and interferon gamma (IFN)–γ [30,31,32,33,34].

sJIA accounts for about 10% of all forms of juvenile arthritis and is a chronic disease that results in significant morbidity and mortality in children [22,35].

The most significant manifestation of systemic arthritis is its association with macrophage activation syndrome, a secondary disorder of excessive, uncontrolled activation and non-malignant proliferation of T lymphocytes and macrophages, with a state of hypercitokinemia, on which the clinical–biological signs depend [36,37,38].

The underlying cause of the occurrence of chronic rheumatism in the JIA subtypes, including sJIA, is largely unknown. A current concept would be that triggering manifestations in sJIA would be due to an infectious aggression with an inappropriate immune response due to a genetic or acquired immune defect [33].

More and more studies have shown that in the pathogenesis of sJIA, the innate immune system is more involved, compared to the adaptive one [31,39,40].

Biological studies for sJIA describe a polymorphism of disease-promoting elements encoded by tumor necrosis factor alpha (TNFα), IL-6, IL-10, macrophage migration inhibitory factor (MIF), and IL-1 family (in particular, IL1A, IL1RN, IL1R2) [41,42,43,44].

More and more clinical and biological as well as translational research draw attention to the particularly important role of IL-1β, IL-6, and IL-18 in the complexity of disease manifestation and the limited role of TNF-α, as well as the relative absence of induced chemokines by interferon gamma (IFN-γ), IFN-γ-inducible protein 10 (IP-10, CXCL10), MIG, and I-TAC [31,40,45].

The IL-1 superfamily contains 11 cytokines, from which IL-1α and IL-1β have the most powerful pro-inflammatory effect. They have a natural antagonist IL-1Ra (IL-1 receptor antagonist), which is an endogenous inhibitor of IL-1 and works as a competitive inhibitor of IL-1 binding to IL-1R1. It is produced by inflamed synovial macrophages and initiates inflammatory responses [46].

IL-1 has a biphasic role in implicating innate immune mechanisms, but also in adaptive ones in triggering sJIA. At the onset of the disease, the disruption of immunity induced by IL-1 induces clinical manifestations of fever, rash, and early synovitis. Then, IL-1 intervenes in the mechanisms of adaptive regulation by activating and promoting the differentiation of T lymphocytes in Th17 cells with a pro-inflammatory role and by inhibiting the activity of T-regulatory cells [47].

Certain evidence of IL-1 involvement in the pathogenesis of sJIA is given by the successful treatment with IL-1 inhibitors, such as the biological agent anakinra, a soluble IL-1 receptor antagonist (IL-1Ra) that is similar to IL-1Ra, which has increased levels during the active disease observed also in polyarticular JIA. At the same time, with particularly good clinical results for anti-IL-1 therapy, the normalization of genes expressed by peripheral blood mononuclear cells (PBMCs) was observed [48,49,50].

Since the response to the IL-1β antagonist therapy of the anakinra product is limited by the large number of IL-1β receptors expressed on several different cell types and the rapid excretion of IL-1RA by healthy kidneys, two drugs (rilonacept and canakinumab) have appeared much more efficient [51].

However, not all patients with sJIA respond well to anti-IL-1 therapy. Clinical–biological research has shown that age at onset of disease, duration, number of active joints, polymorphonuclear, and serum ferritin levels can predict anti-IL-1 response. Elevated ferritin in patients’ serum can be a valuable biological signal and parameter, which will guide us to explore macrophage activation syndrome (MAS), which can be effectively treated with anti-IL-1 agents. So far, the laboratory data have not revealed the presence of antibodies, and there is evidence that sJIA is actually a form of autoinflammatory disease, and not autoimmune [52].

The importance of IL-6 in the pathogenesis of sJIA has been demonstrated by correlating the serum and synovial concentration with the severe joint manifestations and the maximum level of fever [53].

Pro-inflammatory cytokines, such as IL-1, IL-6, and TNF-α, by stimulating similar receptors, induce IL-6 production in lymphocytes, macrophages, and synovial cells [54].

IL-6 has various functions in the pathogenesis of sJIA in children and rheumatoid arthritis in adults, in the sense that it induces an acute phase response as well as activates immune reactions and hematopoiesis.

The released IL-6 will induce the production of acute phase proteins (C-reactive protein and fibrinogen), which are known as biological markers of inflammation and the differentiation of naive T cells into Th17 cells [54,55].

The imbalance between Th17/Treg, where Th17 is activated significantly more than Treg, has a disastrous effect on RA development [56].

As IL-10 is a cytokine that would play a key anti-inflammatory role in the prevention of immune cascades from immune-mediated inflammatory diseases, conflicting results are reported in the literature regarding the poor involvement of this cytokine and the occurrence of manifestations in sJIA [43,44,57].

In a study published by Imbrechts et al., experimental evidence was provided on a mouse model that there would be a relationship between IL-10 insufficient production and its consequence in the innate cellular immune response from sJIA pathogenesis [58].

Both sJIA and macrophage activation syndrome are triggered by a cascade discharge of some cytokines such as interleukin 1β, IL-6 and IL-18.

To date, the exact role of interferon-gamma (IFN-γ), a cytokine with pro- and anti-inflammatory properties is being intensively investigated along with the role of NK cells providing IFN-γ [59].

Put K. found in its published PhD thesis on mice models that “the inflammatory environment in sJIA affects NK cells, causing an inflammatory transcriptional profile in these cells”; although there are very high plasma levels of IL-18, they do not influence NK cells, which causes IFN-y production to be low. Therefore, NK and IFN-y cells should be considered as limiting factors in sJIA pathogenesis [60].

Despite all previous knowledge that IL-18 is commonly recognized as the major inducer of IFN-y synthesis in NK cells, the paper published by Put K. et al. in patients with active systemic JIA shows that despite high plasma levels of IL-18, IFN-y levels remained low. In contrast, gene expression profiling was altered by the increased expression of innate genes, including TLR4 (Toll-Like Receptor 4) and S100A9 (S100 calcium-binding protein A9), and the decreased expression of immunity-regulating genes, such as IL-10RA (interleukin 10 receptor, alpha) and GZMK (granzyme K), as compared to cells from healthy controls. From these studies, it is believed that subtle defects in the pathways associated with NK cells, such as granzyme K expression and IFN-γ production determined by IL-18, may contribute to the immune aggregation of this disease [61].

### Macrophages Activation Syndrome

Macrophage activation syndrome (MAS) or hemophagocytic syndrome is a complication of Still disease in children and adults, which can be life-threatening and is considered a subset of hemophagocytic lymphohistiocytosis (HLH) [62,63].

Macrophage activation syndrome generally has an unknown incidence because some forms are expressed by mild subclinical signs, but 10% of patients with sJIA could have a serious, potentially lethal complication [64].

From the biological point of view, MAS is expressed by a defect of the cytolytic pathways with an uncontrolled proliferation of cytotoxic cells and a hypersecretion of pro-inflammatory cytokines—that is, an increase in hematophagocytic T lymphocytes and macrophages that induce a cytokine storm with severe multiorgan injury [65,66].

In MAS, NK dysfunctions, mutations of the UNC13D, PRF1, STXBP2, and RAB27 genes, TLR-9 receptor dysfunction of IFN-γ, and activation pathways of IL-10 and IL-18 were observed [67,68,69].

Clinical symptoms include fever (96% of cases, often persistent), hepatomegaly (70%), splenomegaly (58%), and lymphadenopathy (51%). In 35% of cases, the neurological manifestations could be convulsions, drowsiness, irritability, confusion, headache, and coma. There may also be cardiac involvement even with pericarditis, pulmonary pleural effusion, hematuria, proteinuria, and signs of renal failure. Hemorrhagic manifestations range from purple rash, ecchymoses, gingival or gastrointestinal bleeding, and disseminated intravascular coagulation. MAS laboratory data include decreased ESR, WBC, platelet count and serum fibrinogen; as well as high and extremely high levels of ferritin, D-dimers, liver enzymes, lactate dehydrogenase, triglycerides, with the prolongation of prothrombin time (PT) and partial thromboplastin time (aPTT) [70,71,72].

Today, there are guidelines with diagnostic criteria for HLH, MAS associated with sJIA, or MAS associated with systemic lupus erythematosus. Selecting the right diagnostic criteria is essential for successful therapy [62].

## 3. Comparative Pathogenesis of Rheumatoid Arthritis in Adults and Children

The pathogenesis of RA and JIA is not yet very well known, although there is strong evidence that it involves the components of the immune system, especially T and B lymphocytes, as well as the antibodies and cytokines resulting from this immune conflict [7,73].

As is well known, the immune system has two main branches: the innate components and the adaptive immunity. The cells and receptors of the innate immune system play an extremely important role in rapidly recognizing the foreign infectious agent and initiating a defense response, which is known as pro-inflammatory [74].

In triggering an inflammatory action, innate immune cells—neutrophils, macrophages, monocytes, natural killer cells, dendritic cells (DC) and so on, playing the role of stopping the inflammatory process (infectious)—will inform, initiate, and direct the phenomena of the proliferation and differentiation of adaptive immune cells [74].

In response to the inflammatory aggression of B cells, the β- and γδ-selected T cells from the branch of innate immunity will be stimulated to proliferate and differentiate into cells specific to the functions appropriate to the immunological challenge, and they will eventually die and leave subsets of cells with memory. In rheumatoid arthritis, the activation of a naive T cell departing from the thymus to the lymphoid organs involves coordinated interactions between a number of molecules on the surface of this cell and an antigen-presenting cell (APC), that is, that carries an antigenic peptide derived from the infectious agent noncovalently linked to a major histocompatibility complex (MHC) class I or class II molecule.

When the APC cell is activated, various costimulatory ligands are expressed, allowing the activation, proliferation, and differentiation of T cells [74,75].

The T cells will express a series of inhibitory receptors for a fine regulation of the response appropriate to the inflammatory environment where they were being stimulated. Inhibitory receptors can act in two directions: to limit the costimulatory signaling, as well as to temporarily bind the costimulatory molecule [74].

At the synovial level, as result of inflammation, the differentiation of naive T cells in Th17 cells will occur. It was believed that this immune pathology would be mediated by Th1 cells (the first objectified), but today, the research has evolved, and it has been discovered that, in fact, Th17 cells are considered responsible for the pathogenesis in rheumatoid arthritis [76,77].

Current studies demonstrate that synovial fibroblasts and activated immune cells are directly involved in the production and release of many pro-inflammatory cytokines that play a crucial role in the development and progression of RA [78].

In fact, the characteristic inflammatory process in RA is achieved by the abundance of inflammatory-promoting cytokines, in counterbalance with inhibitory cytokines, intercellular communication, immune responses, and boosting cell movement to territories of inflammatory, infectious, or post-traumatic conflict.

In RA, the cytokines of the immune network are classified into four groups: pro-inflammatory cytokines, inflammatory cytokines in the joints, anti-inflammatory cytokines, and natural cytokine antagonists [79].

After the onset of initial stimuli, the cytokines play an important role in communicating with the components of the immune system at each stage of the pathophysiology of RA.

The release of cytokines, particularly the TNF-α, IL-6, and IL-1, promotes the synovial inflammatory process.

IL-6 binds to cells via a specific receptor complex involving two proteins, IL-6 receptor α and gp130, in order to transmit information. IL-6 receptor α exists into two forms: a transmembrane IL-6 receptor α, or mIL-6R (membrane-bound form of IL-6R), and a soluble IL-6 receptor α, sIL-6R. After IL-6 binds to any IL-6 receptor, the complex formed will induce gp130 activation. All IL-6-type cytokines signal through the gp130/JAK/STAT pathway. The binding of IL-6 to mIL-6R induces anti-inflammatory classic signaling, whereas the binding of IL-6 to sIL-6R induces pro-inflammatory trans-signaling [80,81,82].

Recent clinical studies have shown that patients with RA (adults) or active polyarticular sJIA (children), who did not respond adequately to MTX (methotrexate) and TNF-alpha inhibitors, received an IL-6 inhibitor, for example, tocilizumab in children and sarilumab (in adults), which are biologics that can be more effective [83,84].

Among the pro-inflammatory cytokines at the synovial level, TNF-α is a pleiotropic cytokine produced by several cell types, such as T and B cells, but also by innate immune cells (dendritic, monocyte, neutrophil, mast cells) and has a very important role, because it participates as the main mediator in regulating and training other factors [85,86].

In addition to this role, it is known that TNF-α is associated with bone and cartilage destruction by activating chondrocytes and osteoclasts [87].

TNF-α induces the synthesis and secretion of MMPs (matrix metalloproteinases), which in turn affect the chemokine and cytokine action of MMP-2, MMP-3, MMP-7, and MMP-9, which release TGF beta (Transforming Growth Factor) from the matrix, thus enabling its activation [88].

Therefore, anti-TNF biological therapy has been considered a remarkable breakthrough in the treatment of chronic autoimmune diseases, such as RA and JIA [85].

The interleukin (IL)-1 (family) together with its members (IL-33, IL-36α, β, γ, IL-37, and IL-38), IL-6, and IL-12 superfamilies (IL-27, IL-35) together with the other key cytokines (IL-15, IL-16, IL-17 family IL-17A, IL-17B, IL-17C), the recently cloned cytokine IL-18, IL-32, IL-34, and interferon (IFN)-y, the granulocyte macrophage colony-stimulating factor, are detected in a high concentration in the synovial fluid, but also in the patient’s serum, thus leading to the process of local joint destruction and systemic effects in the rheumatoid arthritis patient [79,89].

More explicitly, IL-1 has 11 pro-inflammatory and anti-inflammatory members, which are chronologically numbered based on their discovery, from the IL-1 first family member 1 (IL-1F1) to IL-1F11. More commonly, they are also known as receptor antagonist IL-1α, IL-1β, IL-1 (IL-1Ra), IL-18, IL-33, IL-36α, IL-36β, IL-36γ, IL-36Ra, IL-37, and IL-38 [90].

IL-33 has been detected in high serum concentrations in adult patients with rheumatoid arthritis, in contrast to those with osteoarthritis (OA) and psoriatic arthritis (PsA) and was associated with bone erosion and cardiovascular pathology, as a predictive factor for the evolution of atherosclerotic plaque [89]; however, the results are contradictory for its real role in the pathogenesis of RA, and as a consequence, specific drugs are not yet available [91,92,93,94].

Interleukin (IL)-17A is a pro-inflammatory cytokine that participates in the development of several autoimmune and inflammatory diseases [95].

IL-17A has a direct influence on the early pathogenesis and chronic stages of synovitis in rheumatoid and psoriatic arthritis, through systemic, but also local effects on keratinocytes [96].

The synovial membrane in RA is modified by inflammatory factors in the sense of the appearance of a local infiltrate of immune cells, hyperplasia, and angiogenesis tissue [97,98].

It has been shown that in the synovial lymphocyte infiltration and in the hyperplastic mucosa of RA, there are cells producing IL-17A and IL-17F; at the same time, there is a recruitment of Th17 cells that will interact with local cells and perpetuate chronic inflammation [99].

IL-17 is directly involved in the stimulation of vascular endothelial growth factor production in synovial fibroblasts, angiogenesis, and synovial pannus development [100,101].

The interaction between Th17 cells and synoviocytes is crucial, because as a result of this cooperation, IL-17 will be massively released [96].

TNF-alpha supports the effect of human IL-17A for the action of increasing the secretion of IL-6 and IL-8 from rheumatoid synoviocytes and vice versa, IL-17A and IL-17F induce TNFα receptor II expression and production [96,102,103].

Another particularly important role of IL-17 is to promote the expression of nuclear factor kappa-B (NF-κB) ligand receptor activator (RANKL) on osteoblasts and synoviocytes and to activate RANK signaling in osteoclasts [104,105,106].

Since 1999, it is known that IL-17 from human T cells activated in synovial tissues of patients with rheumatoid arthritis is a potent stimulator of osteoclastogenesis [107] and ultimately, the destruction of the bone [96].

The pro-inflammatory cytokines are also responsible for the synthesis of chemokines from MMPs, inducible nitric-oxide synthase, osteoclasts differentiation, and an increased expression of cell adhesion molecules. Disruption of MMP activity can lead to tissue degradation associated with inflammation in rheumatoid arthritis. Several inhibitors capable of modifying MMP activity are approved today, but unfortunately, they are associated with undesirable side effects [88].

Phytochemicals such as flavonoids, glycosides, lignans, and alkaloids are valuable natural sources for the development of new drugs with efficacy and safety in inhibiting upstream signaling molecules involved in MMP expression [88].

Helper T cells are deeply involved in the pathogenesis of autoimmune diseases, including RA; for example, it has been shown recently that Th17 can move into a “non-classical” class of Th1, with higher pathogenic activity, which is a phenomenon that further complicated the explanation of the pathogenic mechanisms of RA [108].

The same study has shown in patients with early-onset RA but without medication a higher ratio of Th17-derived Th1 cells comparatively to CD161 + Th17 cells, and an inverse correlation between interferon-γ (IFNγ) + Th17 cells, comparatively with the anti-CCP antibodies levels [108].

Today, it is known that Th17 produces the cytokine IL-17 [54], which activates inflammation by stimulating immune cells and at the same time activates osteoclasts by inducing kappa B ligand nuclear factor activator receptor (RANKL) in synovial fibroblasts. This fact opens new horizons for Th17-targeted therapies in order to stop the bone destruction associated with T cell activation [109].

At the same time, Foxp3 is essential for the suppressive function of Treg cells, and as a specific marker of Th17 cells, it accelerates osteoclasts differentiation. In RA, Foxp3(+)CD4(+) T cells are subjected to conversion into TH17 cells, which is mediated by synovial fibroblast-derived IL-6 [110], and meanwhile, IFN-gamma cytokines, IL-4, and cytotoxic T lymphocyte-associated protein 4 (CTLA-4), produced by Th1, Th2, and respectively, Treg, regulate osteoclast differentiation.

In RA, there is an imbalance in the Th17/Treg ratio, where Th17 is activated much more than Treg [111].

It is speculated that TNF inhibitors used in RA therapy reduce the passage of Th17 cells to non-classical Th1 cells, as well as direct inhibit the TNFα [85,108].

Inflammatory synovitis both in RA and JIA provides the image of an imbalance between pro-inflammatory and anti-inflammatory cytokines [IL-10, IL-11, and IL-13], which are insufficient to counterbalance the intensely active inflammatory process. Bone destruction in RA is caused by the effects of osteoclasts and not by the invasion of inflammatory factors directly into the synovium [112].

Synovial inflammatory cytokines [TNFα, IL-1, IL-6, and IL-17] promote excessive synthesis of (RANK)/RANKL (receptor activator of nuclear factor kappa-B ligand) on the membrane of synovial fibroblasts and/or osteoblasts and their differentiation [113,114].

Cartilage destruction is caused by MMPs or ADAMTS (a disinterring and metalloproteinase with thrombospondin motifs) that are produced by chondrocytes, synovial fibroblasts, and synovial macrophages. The JAK/STAT pathway is another signaling pathway for various cytokines and growth factors involved in the pathogenesis of rheumatoid arthritis. JAK is a tyrosine kinase receptor that mediates intracellular signaling through a transcription factor, STAT [115].

Currently, there is already experience with inhibitory drugs for the JAK receptor family; thus, tofacitinib for JAK1, JAK2, JAK3, and Tyk2; and baricitinib, which selectively act on JAK1 and JAK2 and are used in RA therapy [116,117].

Forty years after the discovery of IL-1, the “triggering agent” of the molecular and cellular mechanisms of the appearance and development of RA is constantly being sought as another fascinating field of intense investigation. In recent years, the range of pro- and anti-inflammatory cytokines has expanded rapidly with the identification of new members, and it has been proven to be involved to varying degrees in the pathogenesis of RA. Based on this knowledge, the therapeutic arsenal for RA patients includes monoclonal antibodies, fusion proteins, or antagonists against these molecules [89].

The treatment of rheumatoid arthritis in children and adults first benefits from methotrexate as “gold standard therapy”, and if patients do not respond or experience complications and/or adverse reactions, TNF inhibitors will be given: infliximab, etanercept, adalimumab, golimumab, and certolizumab pegol (for adults). While TNF, IL-6, and JAK inhibitors directly regulate cytokine generation and bioactivity, a new biological product, abatacept (as a selective costimulation modulator), has been shown to inhibit T-cell activation by binding to CD80 and CD86, thereby blocking interaction with CD28. This results in the inhibition of autoimmune T-cell activation that is implicated in the pathogenesis of rheumatoid arthritis [118,119,120].

Although there are more and more biological agents with different mechanisms of action for the treatment of rheumatoid arthritis in children and adults, the results are not as we expected, because there are partial responses or non-responsive patients to these compounds, high therapeutic costs, side effects, and so on; therefore, we must turn our attention to other therapeutic modalities to induce disease remission.

## 4. New Introspections and Perspectives on Photobiomodulation in Arthritis

### 4.1. Photobiomodulation: Short History, Basic Concepts, and Current Applications

**Figure 1:**
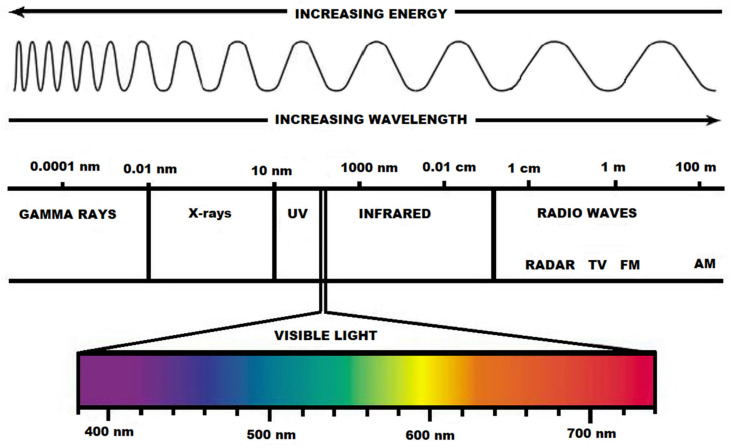
The aspect of the visible light spectrum within the electromagnetic radiation spectrum.

As an interdisciplinary field, photomedicine is growing in importance because of its relevance to light and laser therapies [121]. The whole spectrum of electromagnetic radiation is depicted in Figure 1.

**Figure 2:**
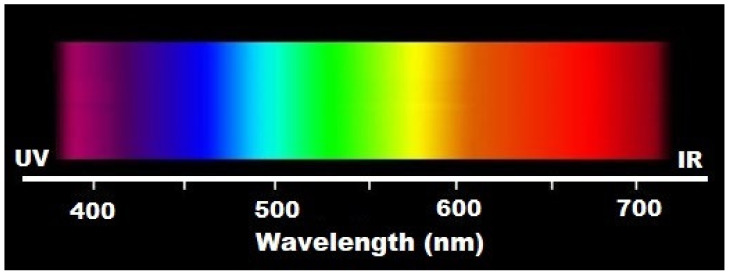
Wavelengths applied in photobiomodulation.

Full-spectrum light or sunlight [122] covers the electromagnetic spectrum from infrared to near-ultraviolet, or all wavelengths that are useful to plant or animal life (Figure 2). Natural light is composed of various electromagnetic waves traveling in disoriented fashion, which is known as incoherent light.

Phototherapy is rooted in the past when Egyptian, Indian, Chinese, and later Greek civilizations used light as a therapeutic agent to cure psoriasis, rickets, vitiligo, and even skin cancers [123].

Since antiquity, we have known that the doctor’s presence is needed where the Sun is missing. Although the therapeutic properties of light have been known for thousands of years, this therapy has been developed and applied extensively only in the last two centuries. For example, the Nobel Prize in Physiology or Medicine 1903 was awarded to Niels Ryberg Finsen “in recognition of his contribution to the treatment of diseases, especially lupus vulgaris, with concentrated light radiation, whereby he has opened a new avenue for medical science” [124].

Lighting with wavelengths ranging from near-ultraviolet to red and including near-infrared has demonstrated many beneficial effects of the stimulation, preservation, and regeneration in cells, tissues, and organs in animals and humans.

After the Nobel Prize was awarded in 1964 to researchers Townes, Basov, and Prohorov for their contributions to the development of laser-maser, applications of low-level laser therapy (LLLT) in multiple branches of medicine have spread around the world, and today, this method is called photobiomodulation (PBM).

LASER (the acronym for Light Amplification by Stimulated Emission of Radiation) was a pure invention of the human mind, which triggered a revolution.

**Figure 3:**
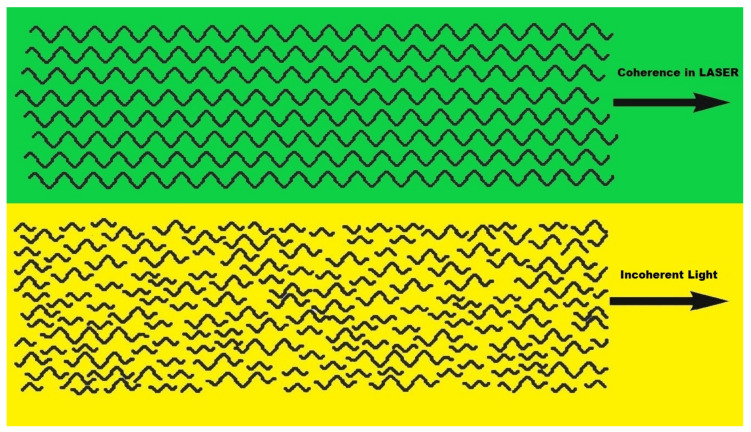
Coherent and incoherent light.

Laser light differs from sunlight due to its three distinct properties: monochromaticity (extremely narrow wavelength range); collinearity (all quanta move into the same direction); and coherence (parallel phase run of the light waves). For example, the difference in the coherence of laser light compared to a lamp is shown in Figure 3.

The most important and useful units of measurement in laser practice are: wavelength (nm, nanometer); power (W, watt or mW, milliwatt); energy (J, joule); power density (W/cm2), and energy density (J/cm^2^).

Light-based treatment methods use lasers or other light sources, such as LEDs; lamps with polarized light, polychromatic, incoherent, and low energy; Super Luminous Diodes (SLD); flash lamps, etc.

These devices release energy into the irradiated tissue that will induce photophysical and photochemical reactions at different biological levels, implicating endogenous chromophores.

There are significant differences between lasers and other light sources, including the specificity of the wavelength and the physical characteristics of the generated beam.

The three unique properties of the LASER beam—monochromaticity, coherence, and collimation—which make it unique for stimulating chromophores in biological tissues that respond only to certain extremely specific wavelengths.

The depth of penetration is determined by the wavelength, the tissue composition, as well as forward and backscatter in the tissue. Coherence is very quickly lost, and the depth of penetration for a large spot (illumination area) can be substantially greater than for a smaller spot size with the same wavelength at the same irradiance (intensity) [125].

If we use PBM with LEDs, there are certain differences, among which we mention lower power delivered in a certain biological time window (for an optimal cellular response); longer wavelength band (approximately 20 nm width), compared to LASER (approximately 1 nm width); the beam is not collimated or coherent [126].

To obtain the desired photobiomodulation effect, a certain quantified amount of photonic energy is always required, and therefore, depending on the pathology, LASERs, LEDs, or other available lamps or devices can be used accordingly.

The success of therapy depends on the correct choice of a device for energy levels quantification targeting.

Today, PBM is widely used worldwide in a variety of pathologies in adult and pediatric medicine. It is a natural treatment that provides the living cells with an energy source in the form of photons. Many diseases or dysfunctional problems of a bodily system or organ can be successfully treated with this ingenious technology. Clinical practice and the scientific investigation had shown bright prospects for the further development of this trend. Lasers can be used to perform exceedingly small and delicate tasks inside the living organisms [127].

Photobiomodulation represents a good option, as it is highly effective in many children’s and adult’s disorders, offering cost-effective advantages over drug therapy, with a quicker more positive response to treatment and no side effects. Last but not least, PBM is painless and non-invasive [128].

For PBM, laser devices are used that have a low light emission power below 500 mW or less than 0.5 watts (class III), but also lasers with a high power of more than 500 mW (laser “therapeutic window” of approximately 650–1100 nm, class IV). The high-intensity laser (HIL) is used with great success especially for sports injuries (traumatic injuries, musculoskeletal strains, osteoarticular, and spine injuries—lumbar and cervical area) [129,130].

Apart from PBM, lasers with higher powers and low pulse widths are applied in surgery, ophthalmology, dermatology–cosmetology, gynecology, oncology, etc.

**Figure 4:**
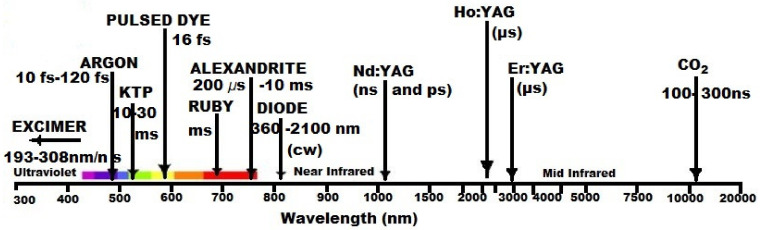
Types of lasers with applications in medicine.

Figure 4 depicts some lasers applied in medicine.

In PBM, from light-emitting diodes or low-level energy lasers, the photonic fluxes enter the cells, penetrating the tissues quite well and initiating a cascade of photochemical reactions on specific signaling pathways due to the endogenous photoreceptors, triggering molecular mechanisms in the mitochondrial respiratory chain, reducing nitrite to nitric oxide, and enhancing the synthesis of the cytochrome c oxidase, which is involved in the electron transport chain in mitochondria [131,132].

Based on more than 30 years of research and treatments in our laboratory, we could really emphasize that PBM is a natural, non-invasive, effective, and well-proved method of treatment for many bodily disorders. Recently, the newest tested technologies such as intravenous, intra-nasal, or sublingual PBM appear to offer the best efficacy for many diseases, from depression to cancer, from acute to chronic pain, for infants and children, to the third age [121,133,134,135].

PBM, as original historical form of governing and influencing life, is able to reset all the body’s self-organizing mechanisms starting from the nucleus to the cellular membranes, and even much more to the cortex and heart, to imprint with information from the millions and millions of the triggered cellular reactions per second in every cell, to balance the internal energy, to normalize the oxygen levels through the two enzymatic reactions of the cytochrome c oxidase, CcO/H_2_O and CcO/NO, as well as to initiate life’s intrinsic mechanisms and the inner biological clock. Even if the whole functional picture is very complex, and some would say that it is still unclear how this form of stimulation might work, we have to think of time in nanoseconds, and it is for sure that the future research will reveal all the cellular and molecular mechanisms underlying PBM [121,136,137].

All the important components of PBM, such as intensity, timing, duration, and wavelength are part of the mainstream process of recovery in a holistic attempt to maximize the benefits of the treatment [121,137].

Being non-invasive and painless, with very few side effects depending on the patient’s health status, and with no known risks associated, PBM heightens the energy, triggering self-organizing phenomena and tissue repair, bringing relief of physical pain or symptoms, and governing the interplay of the oxidative stress by playing multiple roles; it can induce cell proliferation and enhance stem cell differentiation, assisting rejuvenation and normalizing the cellular functions [138,139].

PBM has been proven to target life itself at quantum levels, and so it brings hope for an innovative modulation of immunity, health, and youth. As a practical tool, PBM opens doors for unprecedented and fulminant advances in many nano-medical research fields, by providing the rediscovery of an energetic method for modulating life itself and allowing a systematic generation of data and knowledge through comparison, complementation, and connecting across different medical nanotechnologies [121].

The worldwide tendencies of the current medical fields of the 21st century are innovative energy-based devices and techniques that are highly effective, whether they are drug-free or combined with medication. If professionally managed, the impact on medical practice, especially in Pediatrics, can be revolutionary. The scientific confirmation of these methods is based on discoveries concerning energy and information exchange within living systems, which constitute a ‘‘quantum leap’’ in the understanding and use of light and its interaction with water and other relevant photoacceptors to restore physiologic function [140], cybernetics, biological theory of information, modern thermodynamic concepts, and self-organizing phenomena in complex systems [121].

The advantages of the new energy-based health-care models include the following benefits: they can address the biological processes at their energetic origins; they are able to regulate the biological processes with precision and flexibility; they bring up healing and prevent illness with interventions that can be readily, economically, and non-invasively applied; they include methods that strengthen the immune system; they tend to integrate the body, mind, and spirit, focusing not only on healing, but also on achieving a greater well-being state, especially in patients suffering from chronic diseases [121]; and in the future, they will benefit large, vulnerable population groups, including the elderly and the poor [140], and they will be utilized also at home.

The number of ill children is steadily growing, and they become resistant to some drug preparations starting even from infancy. As a result, new methods for fighting diseases should be figured out. Creative systems and devices, as well as new methods for performing PBM in children for enhanced immunity to fight specific diseases, as for example, juvenile arthritis, do have a great merit and medical value for their capacity to achieve fine-tuned applications, as further interventions for various pediatric diseases, as well as others [141].

### 4.2. Novel Therapeutics Using Photobiomodulation in Arthritis. Where Are We?

A generation ago, children with arthritis faced a lifetime of pain and disability. Juvenile idiopathic arthritis, an umbrella term covering multiple distinct categories, previously called juvenile rheumatoid arthritis until recent reclassification, is one of the most common chronic diseases of childhood, featuring arthritis of unknown etiology [142].

Arthritis with synovial proliferation, triggered by the secretion of pro-inflammatory factors and the formation of granular tissue with monocytes, macrophages, lymphocytes, and other immune cells, will lead to chronic pain and the progressive destruction of the articular structures and functional disability, both in children and in adults [143]. More than one-third of children have ongoing active disease into adulthood with sequelae from chronic inflammation [2,144].

Adult patients and children with moderate or severe forms of arthritis tend to have a worse prognosis, even with the early use of disease-modifying antirheumatic drugs (DMARDs). These patients have considerable morbidity from joint damage, osteoporosis, psychosocial morbidity, reduced quality of life, and educational or employment disadvantage [143,145].

Chronic pain has a large and wholly negative impact on the physical and psychological well-being of patients and their family. Most often, if the inflammation goes away after months or even years of inadequate treatment, the pain may persist for life, due to central sensitization. Childhood chronic pain is a modern public health disaster, which is only now coming to light [2].

In these cases, long-term drugs will induce moderate to severe side effects, so that PBM could be a potential non-invasive anti-inflammatory treatment with minimal side effects [143].

Although the mechanisms of photobiomodulation processes are still being debated, in order to interact with the living cell, light has to be absorbed and has to change the inner cellular state, leading to processes such as the activation of ATP and of protein (RNA, DNA) synthesis, the stimulation of enzyme synthesis, the modulation of prostaglandin synthesis, decrease in the lipid peroxidation rate, the stimulation of specific and non-specific immunity, antioxidant effects, etc. When correctly applied, PBM has the following main clinical effects: improvement of blood circulation and activation of microcirculation, enhancement of collagen synthesis, promotion of tissue regeneration, influence on skin receptors with the increase of pain threshold, improvement of nerve conductivity, acupuncture points stimulation, anti-inflammatory, antiallergic and antiseptic effects, and so on [146].

In children, it is especially valuable because it activates the immunocompetent systems and improves the neurohumoral and hormonal regulation of the metabolism. It must be applied properly and with greater care, because the health problems of children differ from those of adults, and the child’s response to illness and stress varies with age. Each child reacts according to his or her development stage, and to provide the highest quality treatment, the physician requires a familiarity with age-appropriate intervention [126,128].

In certain situations, for an accurate diagnosis and the adequate treatment of infants, children, or adolescents, a multidisciplinary team with pediatric health care experts, as well as key facilities and specific protocols for PBM management of pediatric conditions are needed.

When treating a child with energy-based devices, the physician should have in mind the differences in metabolism, hormonal system, immune system (susceptible to generalized infections, allergic diseases, etc.), and central nervous system (generalization of the post-aggressive reactions in infants and little children). The unique needs of children should be considered by pediatricians and other personnel skilled at evaluating and treating children in such areas as advice, communication, prevention, and therapeutics.

The concept of patient management in infants and children is particularly important. Usually, children are afraid of the physician, or they seem to show a lack of trust toward the doctor and the consulting or treatment area. Furthermore, it is the doctor’s job to cooperate with the child’s parents and to have a supportive attitude to eliminate any kind of stress in the little patient. So, the model on which the doctor–child relation should be built is friendship.

The informed consent is especially important, both in adults and in children. PBM should never be performed on a child if parents or the legal guardians do not fully agree with that. It is even better to allow parents to be nearby the child during the treatment.

The physician should explain to the family and/or the patient that the disease has a chronic evolution, sometimes with little spectacular improvements.

In treating children’s rheumatic conditions, one should have in mind that there are several studies affirming that the usage of PBM on growing articular cartilage may be harmful [147,148].

Consequently, in children’s chronic rheumatic pathology, PBM should be applied by irradiating the blood sublingually, intranasal, venous transcutaneous, or intravenously [149,150,151,152,153]. Sublingual PBM is easy, non-invasive, and with high absorption on intensely vascularized buccal mucosa, triggering rapid systemic effects [134].

The initial approach to the management of patients with rheumatoid arthritis must be vigorous in all patients, to suppress articular inflammation, control systemic disease, prevent secondary deformities, and maintain muscle strength.

The primary aims of treatment in rheumatic pathology include pain relief, preservation of joint function, prevention of deformities, and avoiding drug toxicity. In the long term, minimizing side effects from disease and treatment as well as preserving vision and promoting normal growth and development should be major goals for which PBM can make an important contribution.

Therapy for patients with rheumatoid arthritis should focus on rapid suppression of the inflammatory disease.

The influence of PBM on the immune system has been documented in the medical literature; immunologic effects on leucocytes, T, B, and NK lymphocytes, macrophages, and other cells result in local and systemic effects through a complex mechanism of action that is not fully understood [154].

For a better understanding of concepts and effects, Table 1 presents PBM experimental studies [155,156,157,158,159,160,161,162,163,164,165,166,167,168,169,170,171,172,173,174,175,176,177,178,179,180].

Clinical studies [181,182,183,184,185,186,187,188,189] have also shown that PBM is a promising drug-free tool for inflammatory diseases and arthritis (Table 2).

Aimbire and Albertini et al. demonstrated in an animal model that depending on the dose of PBM, TNF release in acute lung lesions may decrease [190].

Albertini et al. in an experimental study of subplantar muscle in rats used a diode laser with an output power of 30 mW and wavelengths of 660 nm and 684 nm, with the laser beam covering an area of 0.785 cm^2^, at an energy dose of 7.5 J/cm^2^; they proved that COX-2 mRNA expression and edema decreased [191].

Chow R. et al. presented the results of the PBM effect in 16 randomized controlled trials, concluding a pain-reducing effect immediately after treatment in acute forms of neck pain, and up to 22 weeks after completion of treatment in patients with chronic neck pain [192].

Leal-Junior, Lopes-Martins, and Bjordal in a systematic review and meta-analysis of placebo-controlled studies or randomized PBM therapy show that despite growing evidence supporting the value of PBMT in improving and accelerating performance in patient recovery, sample quality needs to be improved to be sure of these effects. They recommend compliance with the Consolidated Test Reporting Guidelines (CONSORT) when designing a research study with PBMT, publishing the protocol with all recommended and used parameters, to allow replication of the study by other authors [193].

Stausholm et al. highlighted in a systematic review and meta-analysis of 22 randomized placebo-controlled trials in patients with pain and disability due to knee osteoarthritis that the pain was significantly reduced in PBM compared with *placebo* at the end of therapy and during follow-up 1–12 weeks later, compared to the placebo group. In addition, the pain decreased (significantly on VAS) at 2–4 weeks after completion of the recommended doses of PBM compared to placebo. There were no reported adverse events. In conclusion, PBM reduces pain and disability in knee osteoarthritis (KOA) at 4–8 J, 785–860 nm wavelength, and at 1–3 J at 904 nm wavelength per treatment site [194].

Following the retrospective evaluation of multiple experimental and clinical studies on the use of PBM on immune cells, appropriate signaling pathways, but also in clinical pathologies, we can support the immunomodulatory effect of PBM and that it is an important complementary and alternative method able to influence the evolution of arthritis and lead to the resolution of joint and systemic inflammatory phenomena through photobiomodulation.

**Figure 5:**
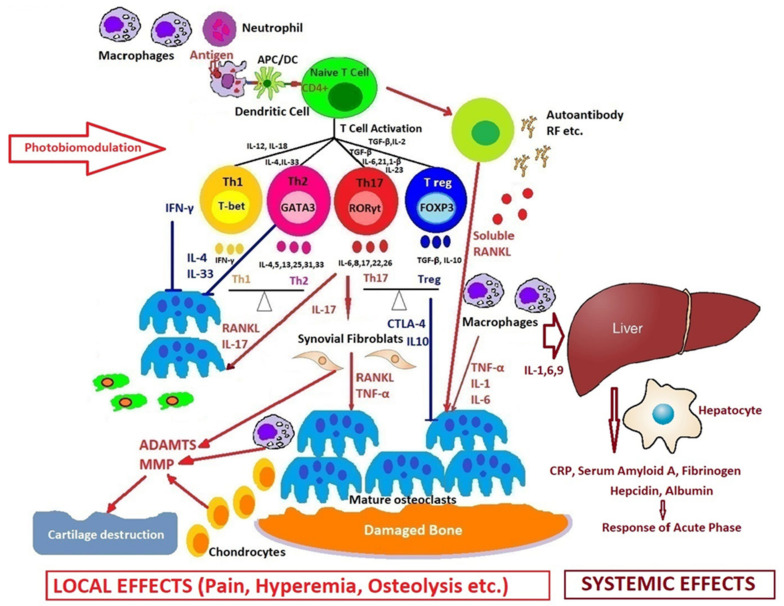
How photobiomodulation could regulate the immune response in arthritis. Possible mechanisms of action on excessive T cell immune response, regulation of pro- and anti-inflammatory cytokines balance, and the process of stopping the proliferative synovium and the osteocartilaginous destruction.

PBM could directly control the autoimmune mechanism by reducing the local and systemic inflammatory response, as in the model we propose in Figure 5.

Figure 5 shows many cells and the resulting cytokines, which participate with different roles in the occurrence and evolution of rheumatoid arthritis. The synovial membrane is penetrated by cells of the immune system (innate and adaptative), and in the synovial fluid appear pro-inflammatory mediators that trigger an inflammatory cascade, activating fibroblast-like synoviocytes and dendritic cells, monocytes, macrophages, mast cells, as well as the T cells and the B cells. An extensive network of new blood vessels is formed, which will lead to the appearance of a synovial hyperplasia, osteocartilaginous erosion, and all the secondary systemic effects.

PBM can very gently modulate the balance between Treg and Th17 cells, i.e., between physiological regulation and the stimulation of the inflammatory process.

An established philosophy in the management of a patient with rheumatoid arthritis is to begin with the safest and simplest therapy judged to be effective. PBM applied in different stages of rheumatoid arthritis is safe, effective, and free of side effects. PBM exerts a positive influence on the synovial membrane and the immune system.

In the inflammatory phase of rheumatoid arthritis, PBM improves the macrophages and lymphocytes activity, decreases the level of immune complexes, and regulates the level of immunoglobulins A (IgA), immunoglobulins M (IgM), immunoglobulins G (IgG), and the balance between pro-inflammatory and anti-inflammatory cytokines [195,196,197,198,199,200].

Photobiomodulation activates the non-specific cellular immune mechanisms, improves the microcirculation of the central and peripheral nervous system, adjusts the functional activity of the hypothalamus and all marginal systems, activates the energetic metabolism, and modulates the immune and vegetative responses. The anti-inflammatory, anti-nociceptive (which elude peripheral and central sensitivity that is the cause for psychological chronic pain) and immunomodulatory effects of PBM allow the reduction, even up to elimination, of the pharmacological drugs and promote the disease remission [201,202].

In recent years, photobiomodulation has become an increasingly mainstream modality, especially in the areas of physical medicine and rehabilitation [130].

Moreover, despite the best efforts of “big pharma,” distrust of pharmaceuticals is growing in general because of uncertain efficacy and troublesome adverse effects. Photobiomodulation has no reported adverse effects, and no reports of adverse events can be directly attributed to laser or light therapy. The high benefit/risk ratio of photobiomodulation should be better appreciated by medical professionals in the rehabilitation and physical medicine specialties [203,204,205,206].

Every patient is unique, and the medical doctor must treat him in an integrative manner for the mind, soul, and physical body.

In the situation of chronic arthritis as in other pathological conditions, a clinician should have a solid knowledge of the disease to be addressed, be up-to-date with its pathogenesis, the modern therapies, and their mode of action, and consider alternatives and complementary therapies that are much older than the pharmacological ones. Only these data should be the basis of the attitude he would have when deciding whether to turn to PBM.

The successful management of PBM in arthritis is based on the clinician’s ability to evaluate correctly the inflammatory status of the patient, to seek the optimal solution, to choose the best technology with the best physical parameters and mode of action, so that the treatment can target very precisely the immune system and the molecular signaling pathways at the molecular level with the exact amount of quantum light energy in order to obtain the desired immune modulation and the remission of the disease.

## 5. Conclusions

Applied PBM could be a safe and an exceptionally good option in the multidisciplinary management of rheumatoid arthritis and chronic pain in children and adults.

To achieve the desired effect of photobiomodulation, a certain quantified amount of photonic energy is always required to target the cells and the immune signaling pathways, to modulate the immune system, and LASERS, LEDs or other available light devices can be used accordingly.

There is currently no consensus on the effective PBM treatment method in improving symptoms and remission of chronic rheumatic diseases.

Successful management of PBM in arthritis is based on the clinician’s ability to evaluate correctly the inflammatory status of the patient, to seek the optimal solution, to choose the best technology with the best physical parameters and mode of action, so that at molecular level the treatment can target very precisely the immune system and the molecular signaling pathways with the exact amount of quantum light energy in order to obtain the desired immune modulation and the remission of the disease.

Light is a very powerful tool in medicine because it can simultaneously target many cascades of immune system activation in comparison with drugs, so PBM can perform very delicate tasks inside our cells to modulate cellular dysfunctions, helping to initiate self-organization phenomena and finally healing the disease.

A lot of information can be stored or transmitted using light.

The near future will be focused on state-of-the-art laser therapy, in an atmosphere concentrated not only on reducing pain and inflammation, but also early healing of the disease.

Interdisciplinary teams should work diligently to meet these needs by also using single-cell imaging devices for multispectral laser photobiomodulation on immune cells.

A new field of innovative research with multiple treatment options in immune-mediated inflammatory diseases opens up by the application of PBM with important clinical implications for the future.

## Figures and Tables

**Table 1:** Experimental photobiomodulation (PBM) studies applied to immune cells and signaling pathways. IL: interleukin, LLLT: low-level laser therapy, MMP: matrix metalloproteinases, NF-kB: nuclear factor kappa-B, TNF-α: tumor necrosis factor alpha.

No	References	Type of Study	PBM Properties	Immune Cells/Signaling Pathways	Brief Results
1.	Castano, A.P.; Dai, T.; Yaroslavsky, I. et al. Low-level laser therapy for zymosan-induced arthritis in rats: Importance of illumination time. *Lasers Surg Med.* **2007**, *39*, 543–550. doi:10.1002/lsm.20516 [155]	Animal Model	810 nm; 5 and 50 mW/cm^2^; 3 and 30 J/cm^2^	Pathway of prostanoids/PGE2	Light regimen (30 J/cm^2^ at 50 mW/cm^2^) effective in reducing swelling of the knees and a greater reduction in the serum PGE2.
2.	Chen, A.C.; Arany, P.R.; Huang, Y.Y. et al. Low-level laser therapy activates NF-kB via generation of reactive oxygen species in mouse embryonic fibroblasts. *PLoS ONE* **2011**, *6*, 22453. doi:10.1371/journal.pone.0022453 [156]	Animal Model	810 nm; different fluences (0.003, 0.03, 0.3, 3, and 30 J/cm^2^); 1 mW/cm^2^ to 30 mW/cm^2^	Murine embryonic fibroblasts/NF-kB	Significant activation of NF-kB at fluences higher than 0.3 J/cm^2^. NF-kB was activated earlier (1 h) by LLLT compared to conventional lipopolysaccharide treatment. Increase in ATP.
3.	Alves, A.C.; Vieira, R.; Leal-Junior, E. et al. Effect of low-level laser therapy on the expression of inflammatory mediators and on neutrophils and macrophages in acute joint inflammation. *Arthritis Res Ther*. **2013**, *15*, R116. doi:10.1186/ar4296 [157]	Animal Model	GaAlAs (808 nm); 50 mW; 0.028 cm^2^ 1.78 W/cm^2^; 4 J; 142.4 J/cm^2^; 80 s/point. 100 mW; GaAlAs (808 nm); 0.028 cm^2^ 3.57 W/cm^2^; 4 J; 142.4 J/cm^2^; 40 s/point	Inflammatory cells (macrophages and neutrophils); gene expression of IL-1β, IL-6, TNFα.	LLLT with 50 mW was more efficient in modulating inflammatory mediators (IL-1β, IL-6) and inflammatory cells (macrophages and neutrophils).
4.	Assis L.; Moretti, A.I.; Abrahão T.B.; de Souza, H.P.; Hamblin, M.R.; Parizotto, N.A. Low-level laser therapy (808 nm) contributes to muscle regeneration and prevents fibrosis in rat tibialis anterior muscle after cryolesion. *Lasers Med Sci*. **2013**, *28,* 947–955. doi:10.1007/s10103-012-1183-3 [158]	Experimental groups and freezing muscle injury (cryoinjury) Adult male Wistar rats were randomly divided.	808 nm; CW; 30 mW power output, 47 s irradiation time, 0.00785 cm^2^ spot area, dose 180 J/cm^2^, irradiance 3.8 W/cm^2^ and 1.4 J total energy per point.	Myogenic regulatory factors (myoD and myogenin), vascular endothelial growth factor (VEGF), transforming growth factor-beta (TGF-β) 1 and type I collagen mRNA	LLLT improved skeletal muscle regeneration by reducing the injured area, increasing myoD, myogenin, and VEGF gene expression and, simultaneously, reducing TGF-β mRNA and type I collagen deposition in the injured tissue. Therefore, LLLT can increase muscle regeneration markers and reduce scar tissue formation, which should favor tissue repair in muscle injuries.
5.	Hsieh, Y.L.; Cheng, Y.J.; Huang, F.C.; Yang, C.C. The fluence effects of low-level laser therapy on inflammation, fibroblast-like synoviocytes, and synovial apoptosis in rats with adjuvant-induced arthritis. *Photomed Laser Surg.* **2014**, *32,* 669–677. doi:10.1089/pho.2014.3821 [159]	Animal Model	780-nm GaAlAs, 30 mW; spot size 0.2 cm^2^, power density 0.15 W/cm^2^. 30 s and 3 min laser irradiation, total fluences at the lower and higher energy densities (power density×irradiation time) of 4.5 and 27 J/cm^2^ were applied daily for five successive days. The accumulated energies delivered from all sessions were 0.9 and 5.4 J, respectively	β-endorphin (β-ep) and TNF-α; substance P and COX-2	This study determined that the fluence provided by LLLT is one of the factors affecting biochemicals related to pain in the treatment of myofascial pain. LLLT irradiation with fluences of 4.5 and 27 J/cm^2^ at myofascial trigger spots can significantly reduce substance P level in dorsal root ganglion. LLLT with lower fluence of 4.5 J/cm^2^ exerted lower levels of TNF-α and COX-2 expression in laser-treated muscle, but LLLT with a higher fluence of 27 J/cm^2^ elevated the levels of β-ep in serum, DRG, and muscle.
6.	dos Santos S.A.; Alves, A.C.; Leal-Junior, E.C. et al. Comparative analysis of two low-level laser doses on the expression of inflammatory mediators and on neutrophils and macrophages in acute joint inflammation. *Lasers Med Sci*. **2014**, *29,* 1051–1058. doi:10.1007/s10103-013-1467-2 [160]	Animal Model	AsGaAl-type diode laser with a wavelength (λ) of 808 nm LLLT at doses of 2 and 4 J on joint papain-induced inflammation in rats; Mean power output (mW = 50); 50; Spot size (cm^2^) 0.028; Power density (W/cm^2^) 1.78; 1.78; Energy (J); 2 and 4 Energy density (J/cm^2^) 71.4; 142.8 Time per point (s) 40; 80.	Inflammatory cells (macrophages and neutrophils); gene expression of IL-1β, IL-6, and IL-10; and TNF-α	Dose of 2 J is more efficient in modulating inflammatory mediators (IL-1β, IL-6, TNF-α, and IL-10) and inflammatory cells (macrophages and neutrophils) and its effects can be observed by histological signs of attenuation of inflammatory processes.
7.	Torres-Silva, R.; Lopes-Martins, R.A.; Bjordal, J.M. et al. The low-level laser therapy (LLLT) operating in 660 nm reduce gene expression of inflammatory mediators in the experimental model of collagenase-induced rat tendinitis. *Lasers Med Sci.* **2015**, *30*, 1985–1990. doi:10.1007/s10103-014-1676-3 [161]	Animal Model	100 mW, 660 nm, 1 J or 3 J, comparatively.	Gene expression for COX-2; TNF-α; IL-6; and IL-10.	Reduction of important pro-inflammatory IL-6 and TNF-α, at 3 J.
8.	Fernandes, K.P.; Souza, N.H.; Mesquita-Ferrari, R.A.; Silva, D.; Rocha, L.A.; Alves, A.N.; Sousa, K.; Bussadori, S.K.; Hamblin, M.R.; Nunes, F.D. Photobiomodulation with 660-nm and 780-nm laser on activated J774 macrophage-like cells: Effect on M1 inflammatory markers. *Journal of photochemistry and photobiology. B, Biology*, **2015**, *153*, 344–351. doi:10.1016/j.jphotobiol.2015.10.015 [162]	Cells Culture J774 were derived from a BALB/c mouse.	660 nm (InGaAlP diode); 780 nm (GaAlAs diode) laser; CW. Average radiant power: 15 and 70 mW. Beam spot size at target: 0.04 cm^2^. Total radiant energy 0.22 J and 0.16 J	Inflammatory cells (macrophages and neutrophils)/mRNA expression of TNF-α and iNOS; production of TNF-α and COX-2 proteins in M1 J774 cells.	660 nm and 780 nm lasers strongly reduced the mRNA expression of TNF-α and iNOS and down-regulated the production of TNF-α and COX-2 proteins in M1 J774 cells.
9.	Assis, L.; Milares, L.P.; Almeida, T.; Tim, C.; Magri, A.; Fernandes, K.R.; Medalha, C.; Muniz Renno, A.C. Aerobic exercise training and low-level laser therapy modulate inflammatory response and degenerative process in an experimental model of knee osteoarthritis in rats. *Osteoarthritis and Cartilage*, **2016**, *24*, 169–177. doi:10.1016/j.joca.2015.07.020 [163]	Animal Model	Diode laser (GaAlAs) 808 nm, cw, 50 mW output power, 28 s irradiation time, 0.028 cm^2^ spot area, 50 J/cm^2^, 1.7 W/cm^2^, 1.4 J total energy per point/section. 3 days/week, at two points on left knee joint, contact technique, for 24 sessions (8 weeks).	IL-1, Caspase-3, and MMP-13 expression in nucleus of chondrocytes	808 nm laser prevented articular degenerative morphological modifications and modulated inflammatory process in OA rats.
10.	Al Musawi, M.S.; Jaafar, M.S.; Al-Gailani, B.; et al. Effects of low-level laser irradiation on human blood lymphocytes in vitro. *Lasers Med Sci* **2017**, *32*, 405–411. doi:10.1007/s10103-016-2134-1 [164]	Irradiation on human blood lymphocytes in vitro	Diode pump solid state (DPSS) laser, with wavelengths of 405, 589, and 780 nm, with an output power of 10 mW and irradiance rate fixed at 30 mW/cm^2^.	Effect of laser at peripheral blood lymphocyte subsets	The effect of laser irradiation fluences of 36, 54, 72, and 90 J/cm^2^ for each wavelength of 405, 589, or 780 nm, with an output power of 10 mW on human blood lymphocyte count in vitro are: no significant differences in lymphocyte count were observed before and after irradiation with the above fluences at wavelengths of 405 and 780 nm; however, a laser wavelength of 589 nm was associated with a significant increase in the lymphocyte count at a radiation fluence of 72 (by 1.6%) and increase in the NK cell lymphocyte subset.
11.	Baek, S.; Lee, K.P.; Cui, L.; et al. Low-power laser irradiation inhibits PDGF-BB-induced migration and proliferation via apoptotic cell death in vascular smooth muscle cells. *Lasers Med Sci* **2017**, *32*, 2121–2127. doi:10.1007/s10103-017-2338-z [165]	Animal experiment In vivo vascular smooth muscle cells (VSMCs)	Low-power laser (LPL) green diode laser 532-nm pulsed wave of 300 mW at a spot diameter of 1 mm.	Apoptosis, migration, and proliferation in vascular smooth muscle cells (VSMCs)/ Caspase-3, Bax, and p38 mitogen-activated protein kinase in PDGF-BB-treated VSMCs.	The study demonstrated that 532 nm LPL irradiation inhibited VSMC proliferation and migration in response to platelet-derived growth factor (PDGF)-BB. LPL irradiation induced apoptosis and enhanced activation of caspase-3, Bax, and p38 mitogen-activated protein kinase in PDGF-BB-treated VSMCs. Based on these results, 532 nm LPL irradiation may inhibit PDGF-BB stimulated proliferation and migration, likely resulting from apoptosis associated with the interaction between 532 nm LPL irradiation and PDGF-BB in VSMCs. Therefore, this study provides a foundation for therapeutic strategies for vascular restenosis via 532 nm LPL irradiation as an alternative treatment against restenosis.
12.	Dos Anjos, L.M.J.; da Fonseca, A.S.; Gameiro, J.; de Paoli, F. Apoptosis induced by low-level laser in polymorphonuclear cells of acute joint inflammation: comparative analysis of two energy densities. *Lasers Med Sci.* **2017**, *32*, 975–983. doi:10.1007/s10103-017-2196-8 [166]	Animal Model/randomly distributed	830 nm, output power 10 mW, 0.05 cm^2^ laser beam area, power density 0.2 W/cm^2^, energy densities:3 and 30 J/cm^2^ (total energy of 150 and 1500 mJ were delivered after 15 and 150 s, respectively), in continuous wave emission mode.	Apoptotic cells in mouse ankle joint samples/DNA fragmentation rate of inflammatory cells Gene expression of proteins involved in apoptosis pathways Bcl2 protein and mRNA expression in PMN cells	The higher energy density (30 Jcm−2) can reduce the inflammatory process by PMN apoptosis induction, while the lower energy density (3 Jcm−2) could also induce apoptosis in PMN; however, this process seems to be slower. The results suggest that apoptosis in PMN cells comprises part of LLLT anti-inflammatory mechanisms and could be a consequence of the balance alteration between expression of proapoptotic (Bax and p53) and anti-apoptotic (Bcl-2) proteins in these cells.
13.	Assis, L.; Tim, C.; Magri, A. et al. Interleukin-10 and collagen type II immunoexpression are modulated by photobiomodulation associated to aerobic and aquatic exercises in an experimental model of osteoarthritis. *Lasers Med Sci* **2018**, *33*, 1875–1882. doi:10.1007/s10103-018-2541-6 [167]	Study of the experimental animals. The degenerative process related to osteoarthritis (OA) in the articular cartilage in rats.	Diode laser GaAIAs 808 nm; CW; power output 50 mW; irradiance 1.7 W/cm^2^; spot area 0.28 cm^2^; dose 50 J/cm^2^; total energy 1.4 J per point/section. Irradiation time: 28 s; local: 2 points (medial and lateral side of the left knee joint) Technique: punctual contact	Chondrocytes/IL-10 expression; transforming growth factor beta (TGF-β) expression; collagen type I (Col I) and II (Col II).	PBM associated with aerobic and aquatic exercise were effective in promoting chondroprotective effects and maintaining the integrity of the articular tissue in the knees of OA rats. PBM and aerobic exercises produced an increase in the expression of TGF-β, which is a member of a superfamily of cytokines; increased Col II and IL-10 expression, which may interfere in cell abnormal metabolism, preventing the matrix degradation and OA progression.
14.	Mergoni, G.; Vescovi, P.; Belletti, S. et al. Effects of 915 nm laser irradiation on human osteoblasts: a preliminary in vitro study. *Lasers Med Sci* **2018**, *33*, 1189–1195. doi:10.1007/s10103-018-2453-5 [168]	A primary culture of human osteoblasts was isolated from mandibular cortical bone of a young health donor.	915-nm GaAs diode laser in the different samples, was administered at 5, 15 and 45 J/cm^2^ with a power output of 1.5 W in continuous wave. Using two different power densities: 0.12 and 1.25 W/cm^2^. Irradiation time was 41.7, and 375 s using a power density of 0.12 W/cm^2^ and 4, 12 and 36 s using a power density of 1.25 W/cm^2^.	Osteoblast proliferation Osteoblast differentiation (bone nodule production)	Osteoblasts treated with a single irradiation per day for 3 days at doses of 5, 15, and 45 J/cm^2^ (power density: 0.12 W/cm^2^ showed no significant differences in terms of cell count compared to controls. PBM at parameters tested in the present study positively modulated the mineralization process in human osteoblasts, inducing the formation of a greater amount of bone nodules but did not increase cell proliferation.
15.	Shakir, E.A.; Rasheed Naji, N.A.; In vitro impact of laser irradiation on platelet aggregation. *Lasers Med Sci.* **2018**, *33*, 1717–1721. doi:10.1007/s10103-018-2527-4 [169]	In vitro blood platelets from 30 healthy volunteers	532 nm; power 100 mW; CW; 4-mm-diameter irradiation beam spot. Irradiation times: 1.8, 3.7, and 6.2 s giving doses of irradiation 1.5, 3, and 5 J/cm^2^, respectively. The divergence was <1.5 m rad, the crystal type of this source was Nd:VYO4:KTP, the laser spot diameter was 0.4 cm, and the power density was 796.17 W/cm^2^.	Platelet aggregation response to laser irradiation /ADP/ATP	Low laser irradiation induced significant changes in platelet aggregation in the presence of weak agonists such as adenosine diphosphate (ADP) and epinephrine. PBM has no influence on platelet count; however, it promotes platelet aggregation in response to weak agonists, specifically ADP and epinephrine.
16.	De Souza Costa, M.; Teles, R.H.G.; Dutra, Y.M. et al. Photobiomodulation reduces neutrophil migration and oxidative stress in mice with carrageenan-induced peritonitis. *Lasers Med Sci.* **2018**, *33*, 1983–1990. doi:10.1007/s10103-018-2569-7 [170]	Animal Model (28 animals were randomly divided)	904 nm ± 5% Operating mode Pulsed. Frequency 1000 Hz; Pulse duration 100 ns; Peak radiant power 50 W; Average radiant power 50 mW.	Cell migration and oxidative stress, in a model of carrageenan-induced inflammation	Treatment with laser decreased the number of leukocytes, especially the neutrophils, in the PBM group and reduced the concentrations of MDA (malondialdehyde), GSH (glutathione), and NO_3_/NO_2_ (nitrate/nitrite) in the peritoneal fluid.
17.	Amaroli, A.; Ravera, S.; Baldini, F. et al. Photobiomodulation with 808-nm diode laser light promotes wound healing of human endothelial cells through increased reactive oxygen species production stimulating mitochondrial oxidative phosphorylation. *Lasers Med Sci* 2019, *34*, 495–504. doi:10.1007/s10103-018-2623-5 [171]	In vitro Human Endothelial Cells (HECV)	808-nm diode laser light emitted by the flat-top handpiece using 1 W of power energy, 1 W/cm^2^ of power density, single dose of 60 J, irradiation of 60 s, fluence of 60 J/cm^2^, mode CW (corresponding to the measured laser therapy of 0.95 W, 0.95 W/cm^2^, 57 J, 60 s, 57 J/cm^2^). To assess the effect of 808-nm laser light irradiation on cell viability, also longer irradiations were performed (100 s and 150 s) corresponding to a final fluences of 100 J/cm^2^ and 150 J/cm^2^, respectively.	HECV/Oxidative phosphorylation aerobic metabolism of HECV, NF-κB/ROS and NO production in endothelial cells.	The present report demonstrated that the short irradiation of 60 s, by the laser setup of 1 W, 1 W/cm^2^, 60 J, 60 J/cm^2^, CW (real measured energy = 0.95 W, 0.95 W/cm^2^, 57 J, 57 J/cm^2^, CW), of HECV in vitro with 808-nm diode laser light was able to stimulate endothelial cell proliferation and oxidative metabolism, which resulted in a more efficient wound repair ability; increase in NO; activate NF-κB; NIR treatment is able to increase the aerobic metabolism, enhancing the O2 consumption and the aerobic ATP synthesis.
18.	Felizatti, A.L.; do Bomfim, F.R.C.; Bovo, J.L. et al. Effects of low-level laser therapy on the organization of articular cartilage in an experimental microcrystalline arthritis model. *Lasers Med Sci.* **2019**, *34*, 1401–1412. doi:10.1007/s10103-019-02740-5 [172]	Animal Model	The gallium arsenide laser device AsGa (λ = 830 nm), CW, fluence = 18 J/cm^2^, power = 40 mW, total energy = 0.36 J, beam area = 0.02 cm^2^, by 9 s. The therapies were applied punctually in the right knee patellar region. After 7, 14, and 21 days of treatment, the animals from the three groups were euthanized (xylazine = 20 mg/kg/ketamine = 40 mg/kg associated with cardiac exsanguination) and the knees were removed and processed for structural and biochemical analysis (*n* = 4/experimental time/analysis) of the AC of the femur and tibia.	Morphometric parameters evaluated in the articular cartilage in male rats. Biochemical parameters evaluated in the articular cartilage (Glycosaminoglycans, Hydroxyproline, Non-collagen proteins) Non-collagen proteins	The present study shows that the phototherapy protocol, using AsGa (λ = 830 nm) in the experimental period employed, was able to revert tissue injuries produced by the microcrystalline arthritis (MA) model in young adult rats.
19.	Han, B.; Fan, J.; Liu, L. et al. Adipose-derived mesenchymal stem cells treatments for fibroblasts of fibrotic scar via downregulating TGF-β1 and Notch-1 expression enhanced by photobiomodulation therapy. *Lasers Med Sci.* **2019**, *34*, 1–10. doi:10.1007/s10103-018-2567-9 [173]	Culture of cells	The total surface of the culture dishes was irradiated for 152 s each time; the energy density of the laser was 4 J/cm^2^. The dual model device emitted florida 6 laser beams (beam diameter <5 mm) at a wavelength of 655 nm (± 5%) and 6 laser beams at a wavelength of 635 nm.	Fibroblasts/TGF-β1 and Notch-1 expression Cell proliferation (CCK-8), cell apoptosis (MUSE), and cytotoxicity (LDH) assays	Results obtained from experiments showed that cell culture supernatant of post-PBM, adipose-derived mesenchymal stem cells (ADSCs) has much more potential as a fibrotic treatment of keloid fibroblasts (KFs) and hypertrophic scar fibroblasts (HSFs), and acting by inhibition of the proliferation, migration, and profibrotic genes synthesis via downregulating TGF-β1 and Notch-1 expression.
20.	Tsuka, Y.; Kunimatsu, R.; Gunji, H. et al. Effects of Nd:YAG low-level laser irradiation on cultured human osteoblasts migration and ATP production: in vitro study. *Lasers Med Sci* **2019**, *34*, 55–60. doi:10.1007/s10103-018-2586-6 [174]	In vivo and In vitro was studied a variety of cell types	Nd:YAG laser (wavelength of 1064 nm) for 60 s at 0.3 W (10 pps, 30 mJ). The total energy density was about 10.34 J/cm^2^.	Migration of cultured human osteoblasts; ATP synthesis	This study showed that Nd:YAG laser irradiation (wavelength of 1064 nm, 0.3W, 10 pps, 30 mJ, 10.34 J/cm^2^, irradiation time 60 s) may contribute to the regeneration of bone tissues owing to enhanced osteoblast cell migration. ATP synthesis was significantly increased in the laser irradiation group compared to the control group.
21.	Cardoso, L.M.; Pansani, T.N.; Hebling, J.; de Souza Costa C.A.; Basso, F.G. Photobiomodulation of inflammatory-cytokine-related effects in a 3-D culture model with gingival fibroblasts. *Lasers Med Sci*. **2020**, *35*, 1205–1212. doi:10.1007/s10103-020-02974-8 [175]	Primary cell culture Gingival fibroblast isolation	12 units of laser diode DL-7140-201S, InGaAsP laser. Center wavelength (nm) 780 nm; spectral band width 780 nm ± 5 nm; Operating mode Continuous wave Frequency 1012 Hz to 1015 Hz; Pulse on duration 40 s; Pulse of duration or duty cycle 40 s; Energy per pulse 0.5 J Peak radiant power 0.07 W; Average radiant power 0.025 W. Number and frequency of treatment sessions 1 irradiation per day, over 3 days. Total radiant energy 1.5 J	Cytokine exposure Cell viability Gene expression of collagen type I and vascular endothelial growth factor (VEGF); Synthesis of VEGF, TNF-α, IL-1β	PBM on the selected parameters (0.5 J/cm^2^, 0.025 W, 780 nm) was capable of adequately penetrating the collagen matrix and positively stimulating human gingival fibroblasts (HGF) wound healing-related functions and decreasing TNF-α synthesis, even in the presence of inflammatory challenge. IL-6 and IL-8 decreased cell viability, the synthesis of VEGF, and gene expression of collagen type I. PBM enhanced cell density in the matrices and stimulated VEGF expression, even after IL-6 challenge.
22.	Katagiri, W.; Lee, G.; Tanushi, A.; Tsukada, K.; Choi, H.S.; Kashiwagi, S. High-throughput single-cell live imaging of photobiomodulation with multispectral near-infrared lasers in cultured T cells. *Journal of biomedical optics*, **2020**, *25*, 1–18. doi:10.1117/1.JBO.25.3.036003 [176]	In vitro T cells culture	Two lasers were adjusted from 200 to 400 mW/cm^2^ for 1064 nm and 50 to 100 mW/cm^2^ for 1270 nm at the focal plane. Dual laser irradiation at an irradiance of 400 mW/cm^2^ for 1064 nm and 100 mW/cm^2^ for 1270 nm was monitored using an IR camera (FLIR Systems).	T cells/PBM on T cells and imaging of intracellular calcium levels and ROS generation nitric oxide binding to cytochrome c oxidase/mitochondrial retrograde signaling	A specific combination of wavelengths at low irradiances (250 to 400 mW/cm^2^ for 1064 nm and 55 to 65 mW/cm^2^ for 1270 nm) modulates mitochondrial retrograde signaling, including intracellular calcium and reactive oxygen species in T cells. The time-dependent density functional theory computation of binding of nitric oxide (NO) to cytochrome c oxidase indicates that the illumination with NIR light could result in the NO release, which might be involved in these changes.
23.	Lemos, G.A., Batista, A.U.D., da Silva, P.L.P. et al. Photobiostimulation activity of different low-level laser dosage on masticatory muscles and temporomandibular joint in an induced arthritis rat model. *Lasers Med Sci*. **2020**, *35*, 1129–1139. doi:10.1007/s10103-019-02933-y [177]	Animal Model	(GaAlAs), 830 nm, 30 mW, 0.116 cm^2^, irradiance 0.259 W/cm^2^, CW, divided as follows: LG5: 5 J/cm^2^, 0.6 J, 20 s/session LG10: 10 J/cm^2^, 1.2 J, 40 s /session LG20: 20 J/cm^2^, 2.4 J, 80 s /session Ten sessions, with 48-h intervals.	Pro-inflammatory cells/IL-1β and TNF-α/ Matrix metalloproteinases (MMPs) family MMP 9 and MMP 2 activity	Results suggest that in this experimental model of joint inflammation, PBM can modulate pro-inflammatory mediators, reducing IL-1β and TNF-α concentrations in affected tissues. LLLT doses promoted better organization of articular disc collagen fibers, a greater number of proteoglycans in articular cartilage, increased area and diameter of left lateral pterygoid fibers, reduced latent and active MMP 9 and 2 activity, and lower IL-1β concentration.
24.	Li, K., Liang, Z., Zhang, J. et al. Attenuation of the inflammatory response and polarization of macrophages by photobiomodulation. *Lasers Med Sci*. **2020**, *35*, 1509–1518. [178]	Culture bone marrow-derived macrophages (BMDMs)	GaAlAs; 810 nm, 2 mW/cm^2^, 4 J and 10 J.	Inflammatory cells (macrophages and neutrophils)/NF-κB p65	PBM suppressed the expression of a marker of classically activated macrophages, inducible nitric oxide synthase; decreased the mRNA expression and secretion of pro-inflammatory cytokines, TNF-α, iNOS, and IL-1β; increased the secretion of monocyte chemotactic protein 1; significantly decreased NF-κB p65 expression in the 4J and 10 J PBM groups.
25.	Moreira, S.H.; Pazzini, J.M.; Álvarez, J.L.G. et al. Evaluation of angiogenesis, inflammation, and healing on irradiated skin graft with low-level laser therapy in rats (Rattus norvegicus albinus wistar). *Lasers Med Sci*. **2020**. *35*, 1103–1109 doi:10.1007/s10103-019-02917-y [179]	Animal Model	AlGaInP 660 nm, 30 mW; its local action area of 2 cm^2^. The laser tip was positioned at a 90° angle in contact with the skin at each predetermined point of the graft, and it was kept for 12 s/point in 6 J/cm^2^ dose and 20 s/point in 10 J/cm^2^ dose. The animals were seen for 15 days, being the sessions performed every 3 or 5 days with 6 J/cm^2^ or 10 J/cm^2^ dose. The groups were G1—control; G2—6 J/cm^2^ every 3 days; G3—10 J/cm^2^ every 3 days; G4—6 J/cm^2^ every 5 days; and G5—10 J/cm^2^ every 5 days.	Fibroblasts/skin grafts/the expression of collagen type III the inflammatory response/COX-2 expression/CD31 expression	It is concluded the exhibition of the skin grafts to 6 J/cm^2^ or 10 J/cm^2^ dose every 5 days improved the healing and the modulation of the local inflammation. The results showed the LLLT may modulate the COX-2 expression in G3, when it has lower average; a greater trend of CD31 expression in G1 was observed as well as less expression in G2. The greater collagen type III–green expression was observed in grafts from G4 in association with greater fibroblasts count. However, in grafts from G5, the collagen type I–red expression was better seen. It was possible to deduce the 10 J/cm^2^ dose every 5 days in G5 resulted in the collagen ripeness.
26.	da Silva, J.G.F.; dos Santos, S.S.; de Almeida, P. et al. Effect of systemic photobiomodulation in the course of acute lung injury in rats. *Lasers Med Sci.* **2020**. doi:10.1007/s10103-020-03119-7 [180]	Animal Model	Red light-emitting diode (LED) (660 nm) 100 mW; 5 J/cm; Energy density 5.35 J/cm^2^; Power density = 33.3 mW/cm^2^; Area = 2.8 cm^2^; total energy= 15 J; time= 150 sec.	Inflammatory cells (macrophages and neutrophils)/ myeloperoxidase activity/ (IL) 1β, IL-6, and IL-17.	PBM on the systemic lipopolysaccharide induced acute lung injury, as it reduced the number of neutrophils recruited into the bronchoalveolar lavage, myeloperoxidase activity, and reduced interleukins (IL) 1β, IL-6, and IL-17 in the lung.

**Table 2:** Clinical effects of PBM on various inflammatory pathologies and associated pain.

Authors/year	Type of Clinical Pathology	Type of Laser/ Wavelength (nm)	Mean Output Power (mW)	Energy Density (J/cm^2^)	Power Density mW/cm^2^/Beam Spot Size (cm^2^)	Area/Pulse (ns)	Time (s or min)	Total E (J)	Results
Bjordal, J.M.; Lopes-Martins, R.A.; Iversen, V.V. A randomized, placebo controlled trial of low-level laser therapy for activated achilles tendinitis with microdialysis measurement of peritendinous prostaglandin E2 concentrations. *Br J Sports Med.* **2006**, *40*, 76–80. [181]	Bilateral Achilles tendinitis	GaAs Infrared 904 nm	Peak power 10 W/Freq. 5000 Hz	20 mW/cm^2^	Fluence 20 mW/cm^2^/0.5 cm^2^	1.8 J for each of 3 points along the tendon	200 ns	In total 5.4 J per tendon	LLLT at a dose of 5.4 J per point can reduce inflammation and pain in activated Achilles tendinitis. LLLT may have potential in the management of diseases with an inflammatory component.
Nakamura, T.; Ebihara, S.; Ohkuni, I. et al. Low Level Laser Therapy for chronic knee joint pain patients. *Laser therapy* **2014**, *23*, 273–277. doi:10.5978/islsm.14-OR-21 [182]	35 subjects with of chronic knee joint pain caused by OA- induced degenerative meniscal tear.	Ga-Al-AS: 830 nm ± 15 nm; CW	1000 mW ± 20%	20.1 J/cm^2^/point	-	20.1 J/cm^2^/point	30 s/point	-	This study confirmed that 830 nm diode laser LLLT was an effective treatment for pain related to knee osteoarthritis.
Soleimanpour, H.; Gahramani, K.; Taheri, R. et al. The effect of low-level laser therapy on knee osteoarthritis: prospective, descriptive study. *Lasers Med Sci*. **2014**; *29*, 1695-1700. doi:10.1007/s10103-014-1576-6 [183]	18 patients with knee osteoarthritis	Gal-Al-As: 810 nm; 50 mW pulse; radiation mode F = 3000 Hz, Δt = 200 ns.	80 W	6 J/cm^2^	0.05 W/cm^2^	1 cm^2^	120 s	36 J	Significant reduction of the nocturnal pain, pain on walking and ascending the steps, knee circumference, distance between the hip and heel, and knee to horizontal hip to heel distance at the end of the treatment course.
		890 nm; 30 mW; F = 3000 Hz Δt = 200 ns	peak power = 50 W	10 J/cm^2^	17 mW /cm^2^	1.765 cm^2^	588 s	53.6 J	
Youssef, E.F.; Muaidi, Q.I.; Shanb, A.A. Effect of Laser Therapy on Chronic Osteoarthritis of the Knee in Older Subjects. *J* *Lasers Med Sci*. **2016**, *7*, 112–119. doi:10.15171/jlms.2016.19 [184]	Patients with OA, Group 1 (*n* = 18)	880 nm, 2 times/ week for 8 weeks = 16 sessions;	50 mW, CW	-	-	-	6 J/point for 60 s, for 8 points	48 J in each session	LLLT to exercise training program is more effective than exercise training alone in the treatment of patients with chronic knee OA and the rate of improvements may be dose dependent, as with 6 J/cm^2^ or 3 J/cm^2^.
	Group 2 (n = 18)	904 nm, frequency 700 Hz; 2 times/ week for 8 weeks = 16 sessions-	60 mW	3 J/cm^2^	Peak power 20 WSpot: 0.5 cm^2^	Pulse duration 4.3 ms	50 s per point	27 J per session	
	Group 3 (*placebo* laser) (*n* = 15)	-	-	-	-	-	-	-	
Nambi, S.G.; Kamal, W.; George, J.; Manssor, E. Radiological and biochemical effects (CTX-II, MMP-3, 8, and 13) of low-level laser therapy (LLLT) in chronic osteoarthritis in Al-Kharj, Saudi Arabia. *Lasers Med Sci.* **2017**, *32*, 297–303. doi:10.1007/s10103-016-2114-5 [185]	Thirty-four subjects with knee OA were randomized into two groups: active group (n = 17) and	GaAs super pulsed laser: 905 nm	25 mW	1.5 J per point for 8 points in total.	-	1 cm^2^	60 s	Total dose 12 J	This study provides the evidence of PBM in reduction of pain and the therapy’s ability to inhibit the proliferation of collagen type II C-telopeptide and other proteins MMP-3 (stromelysin), MMP-8 (collagenase-2), and MMP-13 (collagenase-3), making it an ideal treatment for subjects in the later stages of OA.
	*Placebo* (*n* = 17).	-	-	-	-	-	-	-	
Alayat, M.S.; Ali, M.M. Efficacy of class IV diode laser on pain and dysfunction in patients with knee osteoarthritis: a randomized placebo-control trial. *Bull Fac Phys Ther* **2017**, *22*, 40–5. doi:10.4103/1110-6611.209880 [186]	Randomized blinded placebo-controlled trial 50 patients with knee OA	Ga–Al–Ar 808 nm CW 3 sessions /week/for 4 consecutive weeks	1000 mW	Mean power 500 mW	Spot diam = 2 cm; Aria= 3.14 cm^2^	2.14 J/cm^2^	6 min and 17 s per session	150 J	Class IV diode laser combined with exercise was more effective than exercise alone in the treatment of knee OA. PBM combined with exercise effectively decreased pain and WOMAC index (Western Ontario and McMaster Universities Osteoarthritis Index), as compared with exercise alone.
		905 nm pulsed emission, frequency: 1500 Hz. 3 sessions /week/for 4 consecutive weeks	Peak power 25 W	Mean power 54 mW	Spot diam = 2 cm; Aria= 3.14 cm^2^	Scan on 100 cm^2^; Trigg. Points 6.175 J on each point in an average time of 16 s	9 min.	214 J	
Tomazoni, S.S.; Costa, L.; Joensen, J.; Stausholm, M.B.; Naterstad, I.F.; Leal-Junior, E.; Bjordal, J.M. Effects of photobiomodulation therapy on inflammatory mediators in patients with chronic non-specific low back pain: Protocol for a randomized placebo-controlled trial. *Medicine* **2019**, *98*, e15177. doi:10.1097/MD.0000000000015177 [187]	Randomized placebo-controlled trial PBMT with 5 lasers, skin contact	905 ± 1 nm, Super pulsed infrared 3000 Hz	25 W	1,35 J	17.05 mW/cm^2^ 0.44 cm^2^ (spot)	3.07 J/cm^2^	-		This is the first study that will investigate a possible biological mechanism behind the positive clinical effects of PBMT on non-specific chronic low back pain. We strongly believe that this investigation can be helpful in the management of this condition.
		905 ± 1 nm, Super pulsed infrared1000 Hz	Laser shower 12.5 W	0.225 J	2.84 mW/cm^2^ 0.44 cm^2^ (spot)	0.511 J/cm^2^	-		
		640 ± 10 nm,2 Hz	15 mW	2.7 J	0.9 cm^2^	-	-	4 LEDs Red 24.75 J	
		640 ± 10 nm, 2 Hz2 Hz	15 mW	2.7 J	0.9 cm^2^	-	-	4 LEDs Red 24.30 J	
		875 ± 10 nm,16 Hz	17.5 mW	3.15 J	0.9 cm^2^	-	180 s	4 LEDs Infrared idem	
		875 ± 10 nm,16 Hz	17.5 mW	3.15 J	0.9 cm^2^	-	180 s		
Tsuk, S.; Lev, Y.H.; Fox, O.; Carasso, R.; Dunsky, A. Does Photobiomodulation Therapy Enhance Maximal Muscle Strength and Muscle Recovery? *J Hum Kinet.* **2020**, *73*, 135–144. Published 2020 Jul 21. doi:10.2478/hukin-2019-0138 [188]	Randomized double-blinded placebo-controlled trial	GaAlAs: 808 nm, Pulse frequency: 13 kHz	250 mW	-	Average power: 84.5 mW, Beam size: 1 × 4,5 cm^2^ on 3 quadriceps points located in parallel	Pulse width26 μs	Dose rate: 5.07 J/min (1.13 J/cm^2^/ min)	≈150 J overall treatment	Photobiomodulation protocol of irradiation that was used (20 min, 808 nm, energy of 150 J) did not show beneficial effects on quadriceps muscle performance or recovery after induction of fatigue, when applied immediately after exercise.
Tomazoni, S.S.; Costa, L.O.P.; Joensen, J.; Stausholm, M.B.; Naterstad, I.F.; Ernberg, M.; Leal-Junior, E.C.P. and Bjordal, J.M. Photobiomodulation Therapy is Able to Modulate PGE2 Levels in Patients With Chronic Non-Specific Low Back Pain: A Randomized Placebo-Controlled Trial. *Lasers Surg Med*. **2020**, doi:10.1002/lsm.23255 [189]	Randomized placebo-controlled trial PBMT with multiple lasers, skin contact	SE 25TM Super pulsed infrared: 905 ± 1 nm, 3000 Hz	Peak power 25 W	7.5 mW	0.44 cm^2^ (spot)	17.05 mW/cm^2^	3.07 J/cm^2^	1.35 J	Our results suggest that PBMT was able to modulate PGE2 levels. Results are compatible with the hypothesis that modulating inflammation by decreasing PGE2 levels may be one of the mechanisms involved in the effects of PBMT in patients with LBP.
		Laser Shower 4 Super pulsed infrared: 905 ± 1 nm, 1000 Hz	12.5 W	1.25 mW	0.44 cm^2^ (spot)	2.84 mW/cm^2^	0.51 J/cm^2^	0.225 J	
		SE 25TM 4 RED LEDs (nm): 640 ± 10 nm, 2 Hz	-	15 mW	0.9 cm^2^ (spot)	16.67 mW/cm^2^	3 J/cm^2^	2.7 J	
		Laser Shower 640 ± 10 nm, 2 Hz	-	15 mW	0.9 cm^2^	16.67 mW/cm^2^	3 J/cm^2^	2.7 J	
		SE 25TM 4 Infrared LEDs (nm): 875 ± 10 nm, 16 Hz; Magnetic field: 35 mT	-	17.5 mW	0.9 cm^2^/spot	19.44 mW/cm^2^	3.5 J/cm^2^/ 180 s	Total dose/site 24.75 J	
		Laser Shower 4 Infrared LEDs (nm): 875 ± 10 nm, 16 Hz	-	17.5 mW	0.9 cm^2^/spot	19.44 mW/cm^2^	3.5 J/cm^2^/180 s	Total dose/site 24.3 J	
